# Protein-olive oil-in-water nanoemulsions as encapsulation materials for curcumin acting as anticancer agent towards MDA-MB-231 cells

**DOI:** 10.1038/s41598-021-88482-3

**Published:** 2021-04-27

**Authors:** Pankaj Bharmoria, Meena Bisht, Maria C. Gomes, Margarida Martins, Márcia C. Neves, João F. Mano, Igor Bdikin, João A. P. Coutinho, Sónia P. M. Ventura

**Affiliations:** 1 Department of Chemistry, CICECO-Aveiro Institute of Materials, University of Aveiro, 3810-193 Aveiro, Portugal; 2 TEMA, Department of Mechanical Engineering, University of Aveiro, 3810-193 Aveiro, Portugal; 3 Department of Applied Chemistry, Chalmers University of Technology, Kemivägen 4, 412 96 Gothenburg, Sweden

**Keywords:** Cancer, Biomaterials

## Abstract

The sustainable cellular delivery of the pleiotropic drug curcumin encounters drawbacks related to its fast autoxidation at the physiological pH, cytotoxicity of delivery vehicles and poor cellular uptake. A biomaterial compatible with curcumin and with the appropriate structure to allow the correct curcumin encapsulation considering its poor solubility in water, while maintaining its stability for a safe release was developed. In this work, the biomaterial developed started by the preparation of an oil-in-water nanoemulsion using with a cytocompatible copolymer (Pluronic F 127) coated with a positively charged protein (gelatin), designed as G-Cur-NE, to mitigate the cytotoxicity issue of curcumin. These G-Cur-NE showed excellent capacity to stabilize curcumin, to increase its bio-accessibility, while allowing to arrest its autoxidation during its successful application as an anticancer agent proved by the disintegration of MDA-MB-231 breast cancer cells as a proof of concept.

## Introduction

**Figure 1:**
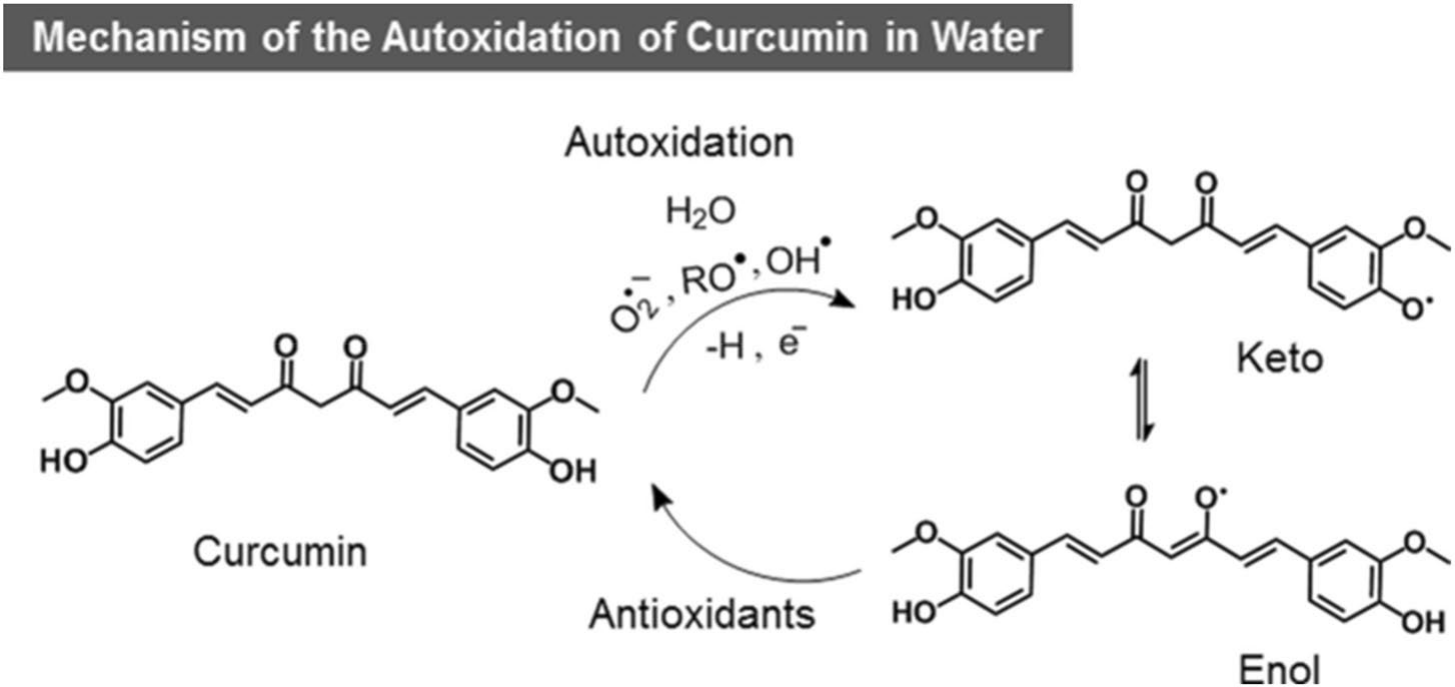
Schematic representation of the autoxidation of curcumin in water to keto and enol radical forms.

Curcumin, a polyphenolic compound present in turmeric, is attracting a lot of interest in recent years due to its potential clinical applications as a nutraceutical^1,2^. Historically, curcumin has been used for centuries as a broad-spectrum medicine in the form of raw turmeric powder in Asia^3^. Recent clinical trials have revealed its pleiotropic activity including antioxidant, antimicrobial, anti-inflammatory and anticancer, thus justifying its medicinal utility since ancient times^4–6^. However, the clinical success of curcumin as a nutraceutical is limited by its poor solubility in water, bioavailability, and bio-accessibility or cellular uptake, as well as its fast autoxidation at physiological pH (t_1/2_ ~ 20 min)^7–10^. Recently, Goto’s research group reported the curcumin enhanced solubility of 8 mg mL^−1^ with t_1/2_ ~ 260 min in its aqueous complex using fatty acid ionic liquids^11^. While fatty acids can protect curcumin from autoxidation, they cause the poor cellular uptake of curcumin^12^ due to their hydrophobic interactions with the fatty acids of the cell membrane. This ultimately cause deep insertion of curcumin into the cell membranes limiting its diffusion to the cytoplasm^2^, explained the authors. Furthermore, other strategies have been studied to overcome these problems, which include the development of micelles and nanoemulsions^13–16^, liposomes and carbohydrate-coated liposomes^17–19^, emulsions and nanoemulsions^20–25^, solid-lipid nanoparticles^26–29^, and very recently, the nano-porous starch aerogels-based curcumin nanoparticles^30^. The McClements’s research group has recently reviewed these strategies and concluded that the reported systems exhibit serious shortcomings like the high manufacturing costs and toxic concentrations of the food grade surfactants used. Moreover, they suggested for protein encapsulation strategy to improve cellular uptake and reduce cytotoxicity. In an interesting report, Jovanovic et al.^31^ have reported that antioxidants soluble in water can assist in regeneration of its autoxidized oxyl radical with electron transfer. Additionally, proteins function as a transporter across the cell membrane in vivo^32,33^ and if we could encapsulate stable curcumin with protein it can easily pass through the semipermeable membrane barrier for cellular uptake. Based on these assumptions, we investigated the encapsulation of curcumin in olive oil coated with protein to prevent curcumin autoxidation and guarantee its stable cellular uptake. Herein, we have developed a new, simple and robust strategy of entrapping curcumin in the confined antioxidant pool of olive oil stabilized by a cytocompatible copolymer, the Pluronic F 127 (PF 127) in water, in the form of nanoemulsions encapsulated by a protein (gelatin) with a structural evidence. It is also the first report on the development of oil-in-water nanoemulsions (NE) of olive oil with PF 127. Gelatin encapsulated nanoemulsions containing curcumin (G-Cur-NE) were tested for cellular uptake by L929 cells and compared with Cur-NE. The G-Cur-NE were also tested for their anticancer activity towards MDA-MB-231 breast cancer cells in comparison to healthy L929 cells as a proof of concept. Autoxidation of curcumin at the physiological pH to deoxygenated bicyclopentadione is the key degradation channel^5,34^. Autoxidation begins with the abstraction of hydrogen atom from one of the phenolic hydroxyls of curcumin by radical scavengers, which can be avoided by placing it in a pool of antioxidants (Fig. 1)^31^.

**Figure 2:**
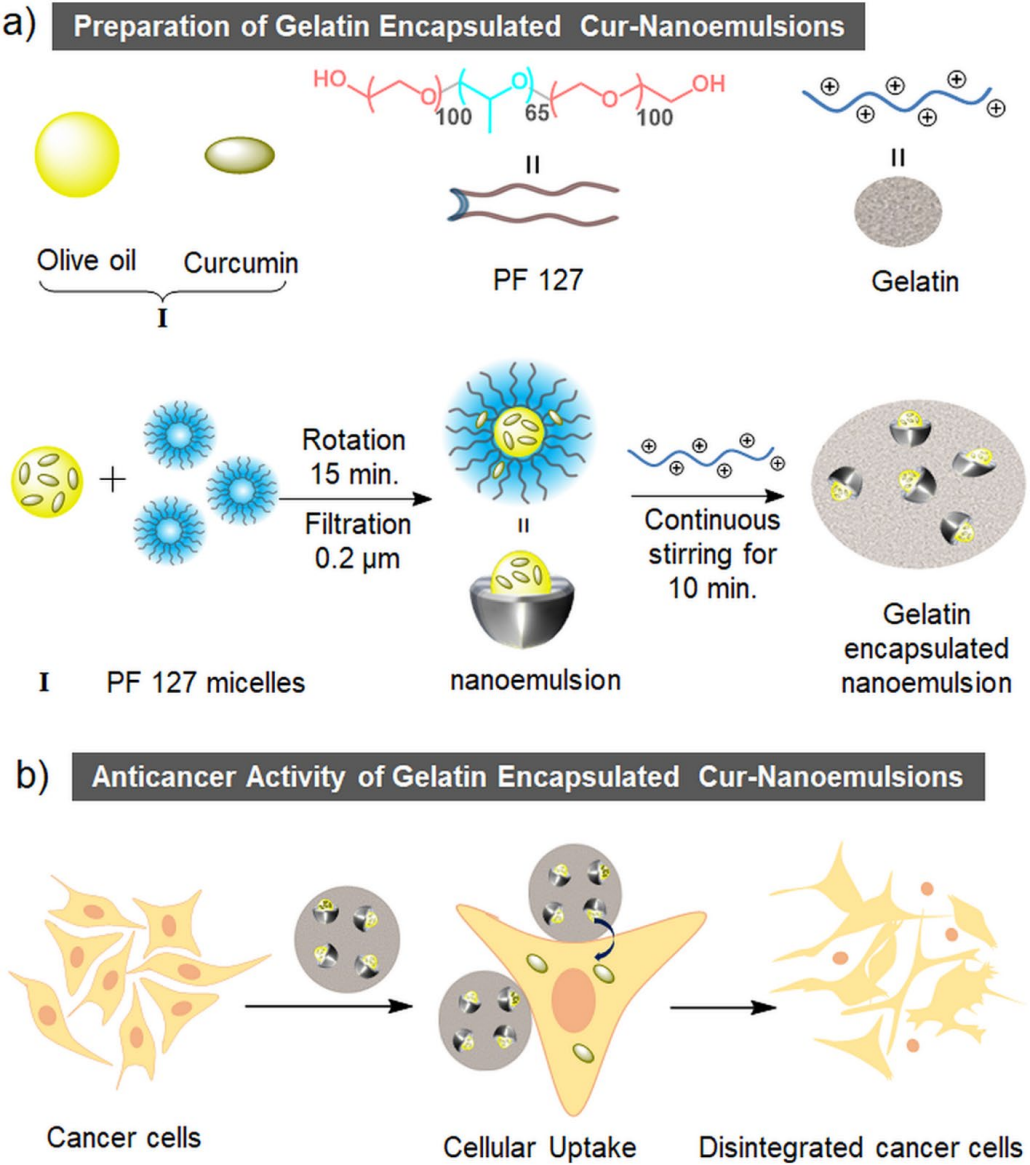
(**a**) Chemical structure of PF 127, model structures of olive oil, curcumin and gelatin; preparation procedure of gelatin encapsulated olive oil-PF 127-water nanoemulsions containing curcumin (G-Cur-NE). (**b**) Schematic representation of the cellular uptake and anticancer activity of G-Cur-NE towards MDA-MB-231 breast cancer cells.

Olive oil is comprised of many antioxidants^35,36^ to trap the radicals like O_2_˙ˉ, RO˙ and OH˙. Therefore, we have used it to avoid the autoxidation of curcumin in aqueous formulations in the form of oil-in-water nanoemulsions. Moreover, due to the presence of high amounts of antioxidants, olive oil can itself show anticancer activity^37^. PF 127 has been used because of its cytocompatible nature since it is commercially used to culture cell lines^38^. Recently, its hydrogel was reported as a biocompatible matrix for NIR light triggered optogenetic behaviour of neurons^39^. Moreover, having a hydrophilic-lipophilic balance (HLB) higher than 16, in theory, the PF 127 shows appropriate properties to form oil-in-water nanoemulsions^40^. Gelatin type A being a positively charged protein^41^ allows the encapsulation of the negatively charged nanoemulsion. Being a protein, gelatin is expected to provide a suitable transit for curcumin to pass through the semipermeable cell membrane by mimicking the carrier proteins^32,33^. In this context, the schematic representation of the preparation of gelatin encapsulated curcumin loaded nanoemulsion is shown in Fig. 2a and its cellular uptake leading to anticancer action on MDA-MB-231 breast cancer cells shown in Fig. 2b.

## Materials and methods

### Reagents

Curcumin, Pluronic F 127, Tween 80 and gelatin Type A, Whatman SPARTAN RC 30 syringe filters with pore size 0.2 μm were purchased from Sigma-Aldrich. The olive oil (azeite virgem extra, SELEÇÃO, Oliveira da Serra) with product number 5601024124083 was purchased from a local supermarket. The oil is comprised of triglycerides and phenolics. The antioxidant activity of olive oil was measured using ABTS assay using literature procedure^42–45^ and was found to be 932 ± 10 µmol Trolox kg^−1^. Methanol and hexane (Analytical grade) wer purchased from Carlo Erba. ABTS (98%) was purchased from Alfa Aesar. Pottassium persulfate (extra pure) was purchased from Scharlau. Trolox (97%) was purchased from Across Organic. KH_2_PO_4_ (> 99.5%) was purchased from Merck and K_2_HPO_4_ (98%) was purchased from Sigma Aldrich. Cell culture media and supplements, namely GIBCO Dulbecco’s phosphate buffered saline (DPBS), fetal bovine serum (FBS; E.U. approved, South America origin), Dulbecco’s modified Eagle’s medium low glucose (DMEM–low Glc), Dulbecco’s modified Eagle’s medium high glucose (DMEM–high Glc), TrypLETM Express, GIBCO antibiotic/antimycotic solution (ATB) containing 10,000 units mL^−1^ of penicillin, 10,000 mg mL^−1^ of streptomycin, were purchased from ThermoFisher Scientific (Alfagene, PT). Adherent 96-well plates were purchased from InVitroCell (Sarstedt, PT), non-treated clear bottom 96-well black plates from Corning (Corning, USA) and 13 mm round-treated coverslip from Sarstedt (Sarstedt, PT). Calcein-AM, Propidium Iodide (PI), DAPI and Fluorescein Phalloidin were all purchased from Thermo Fisher Scientific Inc (Alfagene, PT). All samples were prepared in distilled water.

### Measurement of the antioxidant activity of olive oil

The phenolics present in the olive oil act as a radical scavenger, providing the antioxidant activity (AA) to olive oil. Phenolics were extracted by liquid–liquid phase separation by mixing olive oil with hexane and methanol:water mixture^42^. For this, 4 mL of olive oil was mixed with 2.5 ml of hexane and 2.5 ml of methanol:water mixture (80:20). The mixture was centrifuged for 10 min. at 1000 rpm at room temperature. The bottom phase was separated (sample) and the top phase was mixed again with 2.5 mL of hexane and 2.5 mL of methanol:water mixture followed by centrigation. The process was done in triplicate to extract the maximum amount of phenolics in the bottom phase leading to samples 1 to 3, characterized by having different concentrations in phenolics.

The antioxidant activity was determined by the 2,2-azino-bis(3-ethylbenzothiazoline)-6 sulfonic acid (ABTS) radical cation decolorization assay^42–45^. The ABTS^• +^ was generated by mixing 5 mL of ABTS (7.46 mM) with 5 mL of potassium persulfate (2.5 mM) followed by incubation in dark at room temperature for 12 h. The mixture was then diluted with a potassium phosphate buffer (pH 7.4) until the absorbance at 734 nm becomes 0.70 ± 0.05. Then, 100 µL of the samples 1, 2 and 3 (olive oil extract) were mixed with diluted ABTS^• +^ approaching the final volume of 2 mL. The scavenging ABTS^•+^ radical imposed by the presence of phenolics was monitored from the decease in absorbance at 734 nm for 1 h in a microplate reader. The % decrease in ABTS^• +^ after 30 min was used to calculate the antioxidant activity. The mixture of 4 mL of water + 2.5 mL of hexane + 2.5 mL of methanol:water was used as blank. A calibration curve of aqueous solution of Trolox (0.1 to 0.8 mM) was used to calculate the antioxidant activity in µ mol Trolox kg^−1^. The final antioxidant activity was calculated as addendum of samples 1, 2 and 3 obatined by liquid–liquid phase separation.

### Preparation of olive oil in water nanoemulsions

For nanoemulsion preparation we used 2% of an aqueous solution of PF 127 (1.59 mmol kg^−1^) and added 7.5 mg g^−1^ or 15 mg g^−1^ of olive oil into the PF 127 solution. The addition of PF 127 solution was done at low temperature (4–10 °C) to avoid the creaming. The solutions were ultrasonicated for 5 s followed by homogenization by vortexing at 2500 rotations *per* minute (r.p.m.) for 15 min. Unlike the reported procedures for nanoemulsions formation, only 5 s of ultra-sonication were used, since ultrasonication for longer periods may start the de-mixing of olive oil from the PF 127 micellar solution. Solutions were then allowed to rest for 10 min. for stability verification, followed by filtration with a 0.2 µm membrane filter (Fig. S1a from ESI). A faintly turbid filtered solution was then allowed to rest for 3 h before any further measurements.

### Cell culture and ME incubation

Mouse fibroblasts (L929) and human breast cancer fibroblasts (MDA-MB-231) cells were cultured in DMEM low Glc and high Glc, respectively, supplemented with 1% Penicillin/Streptomycin and 10% fetal bovine serum. Both cell lines were maintained in a CO_2_ incubator with temperature set at 37 °C, and the medium replaced every two days. One day before each assay, the cells were seeded at a density of 1 × 10^3^ and 5 × 10^3^ cells/well on 96-well plates and 8-well IBIDI μ-slide, respectively. The old culture medium was then replaced with a fresh one along with the addition of 33% of newly ME samples prepared as previously described, and cells further incubated for 72 h at 37 °C.

### Cell viability and morphology assay

The cell viability was determined by i) AlamarBlue assay (ThermoFisher Scientific, USA) to assess the metabolic activity and, ii) Live-Dead assay to assess the membrane integrity. The AlamarBlue assay was performed according to the manufacturer’s guidelines. Briefly, 10% of AlamarBlue buffer was added to each well and the incubation allowed for 3 h. Afterwards, aliquots of 100 µL were taken out from each well and transferred to a non-treated 96-well black plates with clear bottom. The fluorescence intensity was measured by a multimode microplate reader. Here, the purple resazurin is reduced to the pink colored resorufin by live cells. The error bars indicated STD from the means of three independent experiments. For the Live/Dead assay, cells seeded on 8-well IBIDI μ-slide were labelled with Calcein-AM (3 µg mL^−1^) and Propidium Iodide (PI) (6 µg mL^−1^) for 30 min at 37 °C. Following incubation, cells were rinsed with DPBS and immediately observed by LSM fluorescent microscopy. Green staining indicates good integrity of the cell membrane and therefore live cells, while red staining indicates membrane disruption and staining of the nucleus by the PI. Cell morphology was evaluated by F-actin staining with flash phalloidin red 594 or flash phalloidin green 488. For that, after incubation with each sample, cells were fixed with 4% formaldehyde during 10 min, rinsed with DPBS and incubated with phalloidin (25 µg mL^−1^) for 30 min at 37 °C.

### Statistical analysis

All statistical analysis was performed using Graphpad Prism 6 software (Prism 6TM). One-way analysis of variance (One-ANOVA) and Two-way analysis of variance (Two-ANOVA) with Holm- Sidak’s post-hoc test. Six replicates were used for statistical analysis. Unless otherwise indicated, *p-value < 0.05 and **p-value < 0.001 were considered statistically significant.

### Characterization of nanoemulsions

For the extraction of phenolics from olive oil, the mixture was centrifuged using a Centrifuge Kubota 2010. The absorbance changes of ABTS^• +^ due to scavenging by phenolics at 734 nm were monitored using a Synergy HT microplate reader, BioTek. The presence of curcumin in olive oil, NE and G-NE was detected from UV–visible spectral measurements using an UV-1800 SHIMADZU UV-spectrophotometer in a quartz cuvette. The spectra of curcumin in olive oil were acquired in a path length quartz cuvette of 0.1 cm upon diluting the samples to 1:10. The spectra of solutions of curcumin in nanoemulsions and gelatin coated nanoemulsion were acquired in 1 cm path length quartz cuvette. All spectra were corrected for baseline with the base solution containing the olive oil. The Zeta potential of nanoemulsion and gelatin coated nanoemulsions using a Zetasizer Nano ZS light scattering apparatus (Malvern Instruments, UK) was measured. The samples were filtered using syringe filters of 0.2 μm. The size of PF 127 micelles, nanoemulsions and G-NE was measured from their hydrodynamic diameter, using Zetasizer Nano ZS light scattering apparatus (Malvern Instruments, UK) with a He–Ne laser (633 nm, 4 mW). The samples were analyzed in a quartz cuvette with 1 cm pathlength at a scattering angle of 173°. Again, the samples were filtered using a syringe filter with 0.2 μm. The Ostwald ripening of micelles with time in NE and G-NE was also measured from their time dependent hydrodynamic diameter using a Zetasizer Nano ZS light scattering apparatus (Malvern Instruments, UK). The mixing of samples for nanoemulsion preparation was done with I@LAB MX-S Rotor Equipamentos de Laboratório, Lda, at its maximum rotation speed (2500 rpm). The surface topology measurments of the PF 127 micelles and NE were acquired from AFM imaging. Images were acquired using the Veeco AFM Multimode Nanoscope (IV) MMAFM-2, USA, in a semi contact mode. For the determination of the AFM imaging, aliquots of 5 μL of PF 127 micelle and NE solutions were deposited onto a carbon-coated copper grid and allowed to dry for 24 h in air. The structure of G-NE was resolved from Transmission Electron Microscopy (TEM) imaging using a Hitachi STEM HD2700 microscope operating at 200 kV. 5 μL aliquots of G-NE were deposited onto a carbon-coated copper grid and allowed to dry for 24 h in air. The cell viability was analyzed from fluorescence intensity of the AlamarBlue assays in a multimode microplate reader (Synergy HTX), equipped with a Tungsten halogen lamp and a PMT detector. Intensities were acquired from the bottom of the well plate by a filter-based fluorescence optics (Ex: 540/35 nm; Em: 600/40) and processed in Gen5TM software. Fluorescent microscopy of the cells to know their living or dead state was performed using a laser scanning confocal microscope (LSM 880 Airysccan, Carls Zeiss, Germany) using 20 × or 40 × objectives. The acquired images were analyzed in Zeiss Zen Blue software | (2017).

## Results

### Preparation and characterization of olive oil-PF 127-water nanoemulsions (NE) and gelatin coated me (G-NE)

**Figure 3:**
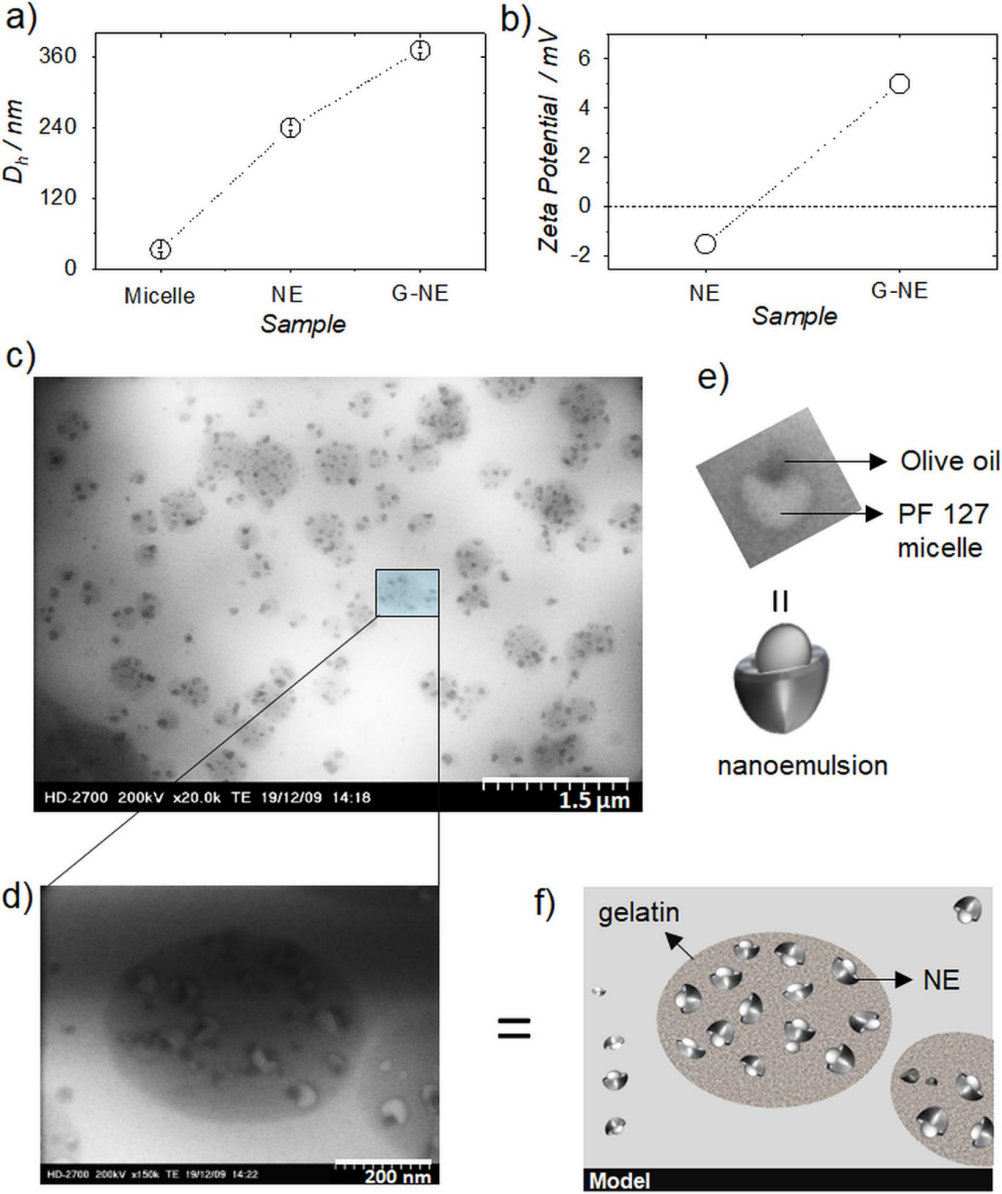
(**a**) Plot showing difference in hydrodynamic radii (Dh) of PF 127 micelle, nanemulsion (NE) and gelatin encapsulated nanemulsion (G-NE). (**b**) Difference in zeta potential of NE and G-NE in solution. (**c**) TEM image of G-NE (scale bar 1.5 µm). (**d**) Cross section TEM image of G-NE showing nanoemulsion encapsulated with gelatin (scale bar 200 nm). (**e**) TEM image along with the model of single NE showing olive oil droplet sitting inside the pocket of PF 127 micelle. (**f**) Model of gelatin encapsulated nanoemulsion.

Firstly, we tested the feasibility of nanoemulsion formation with olive oil, since there are not large number of reports on its NE or microemulsion (ME) formation^46,47^. The detailed procedure is provided in methods section and Fig. S1a from ESI. The formation of a NE was confirmed from the difference in % intensity of hydrodynamic diameter (D_h_) between PF 127 micelles (33 ± 3 nm) and nanoemulsion (240 ± 5 nm), TEM and AFM images as shown in Figs. 3a and S2a, S4 and S5a,b from ESI. The thermodynamic stability *vs* kinetic stability is one of the ways of defining between NE and ME^48^. In this work, the stability of the emulsion was measured and found to be stable up to a month (Figs. S6 and S7 ESI). After this period, the NE started to demixed at room temperature, whereas the materials stored at 4–8 °C remained visually stable, although with an increase in size to 302 nm after three months of storage (Fig. S8 ESI). Therefore, it is kinetically stable and can be categorized as NE^48^. The gelatin encapsulated nanoemulsions (G-NE) were prepared by adding 1.5 mL of 1% of an aqueous solution of gelatin type A to 1.5 mL of filtered nanoemulsion solution with continuous stirring for 10 min. followed by the solution rest for 24 h. (Fig. S1a from ESI). The coating of gelatin around NE’s was confirmed from DLS, zeta potential and TEM imaging (Figs. 3a–d, and S2, S3, S9, from ESI). DLS distributions showed size range of 150–600 nm with a maximum intensity at 372 nm (Figs. 3a and S2c from ESI), whereas positive zeta potential (+ 5.00 ± 0.05 mV) was observed for G-NE solution indicating a positive charge on its surface, due to the encapsulation by gelatin (Figs. 3b and S3b from ESI).

The negative zeta potential (− 1.5 mV) obtained for NE’s (Figs. 3b and S3a from ESI) may be explained by the presence of some phenolics in the olive oil. As referred, olive oil is composed essentially by triglycerides and phenolics. At physiological pH, some of the phenolics present may be negatively charged and considering their structure, they definitely will be present in the surface of the nanoemulsions formulated, interacting with the outer environment, the water, a result already explored in a recent publication^49^ by some of us, where coarse-grained molecular dynamics was applied to rationalize the biomolecules solubilization mechanisms, and for which, the conclusions on the gallic acid behaviour are elucidative.

TEM images of G-NE gave absolute clarity of the encapsulation of NE by gelatin as 1 to 10 NE’s can be seen encapsulated inside the gelatin capsules (Fig. 3c). The size distribution of G-NE in TEM (Fig. 3c) is like that observed from DLS measurements (Fig. S2c, supporting information). At higher resolution, TEM exposed the perfect structure of the nanoemulsion wherein olive oil droplets could be clearly seen sitting in the pocket of PF 127 micelles (Fig. 3d,e). Additional TEM images are provided in Fig. S9 from ESI. A model structure of Fig. 3d is presented in Fig. 3f for better clarity of the structures obtained.

**Figure 4:**
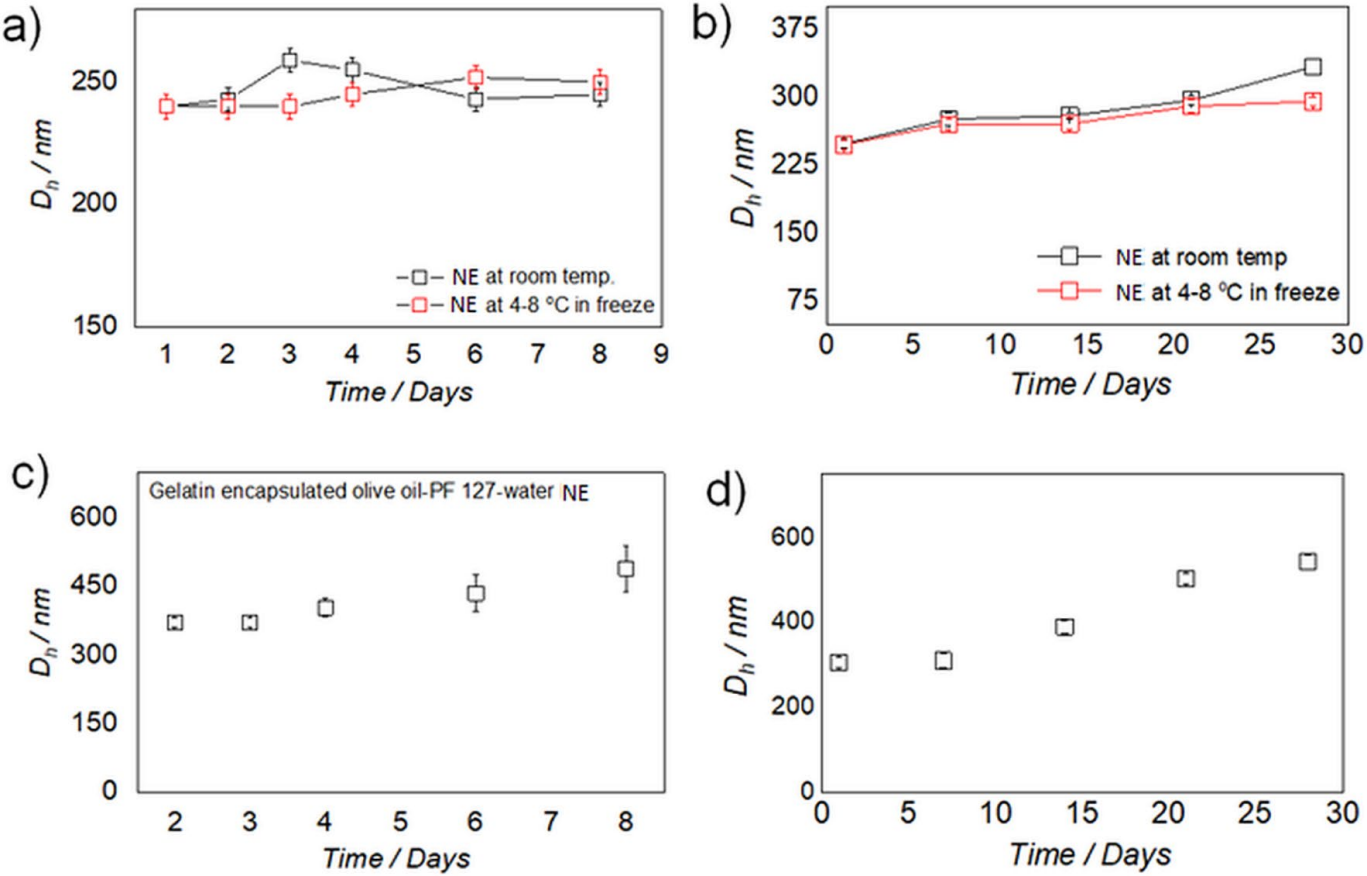
(**a**,**b**) Variations in hydrodynamic diameter (nm) with time (days) of olive oil-PF 127-water nanoemulsions stored at room temperature (black open square) and stored at 4–8 °C in a refrigerator (red open squares). (**a**) for a week and (**b**) for a month. (**c**,**d**) Variations in the hydrodynamic diameter (nm) with time (days) of gelatin encapsulated olive oil-PF 127-water nanoemulsions (G-NE) stored at 4–8 °C in refrigerator, (**c**) for a week and (**d**) for a month.

The stability of both NE and G-NE over time was studied for a week and a month separately, at room temperature and in a refrigerator at 4–8 °C (Figs. 4 and S6, S7 and S10–S11 from ESI). DLS was used to check the increase of the micelle diameter (Fig. S5a,b from ESI). No increase of the micelle diameter was observed for the prepared NE’s, as size distributions were largely retained under the tested conditions for a week (Figs. 4a and S6 from ESI). However, a small ripening effect of micelles was observed when stored for a month at both temperature conditions studied. These results indicate that the NEs remain largely stable in the solution up to a month (Figs. 4b and S7 from ESI). Gelatin being a protein is prone to degrade with time; therefore, the stability of G-NE’s was studied by storing them in a refrigerator from a week to a month. An increase in size of the hydrodynamic diameter of G-NE was observed with time (Figs. 4c,d and S10, S11 from ESI). This can be justified by interactions between the gelatin molecules, as it is a low temperature gelator and the concentration used was just below its gelation concentration. However, this issue could be easily resolved by shaking the solution at room temperature because the hydro-gelation of gelatin is thermo-reversible.

### Stability of curcumin in NE (Cur-NE) and G coated NE (G-Cur-NE)

**Figure 5:**
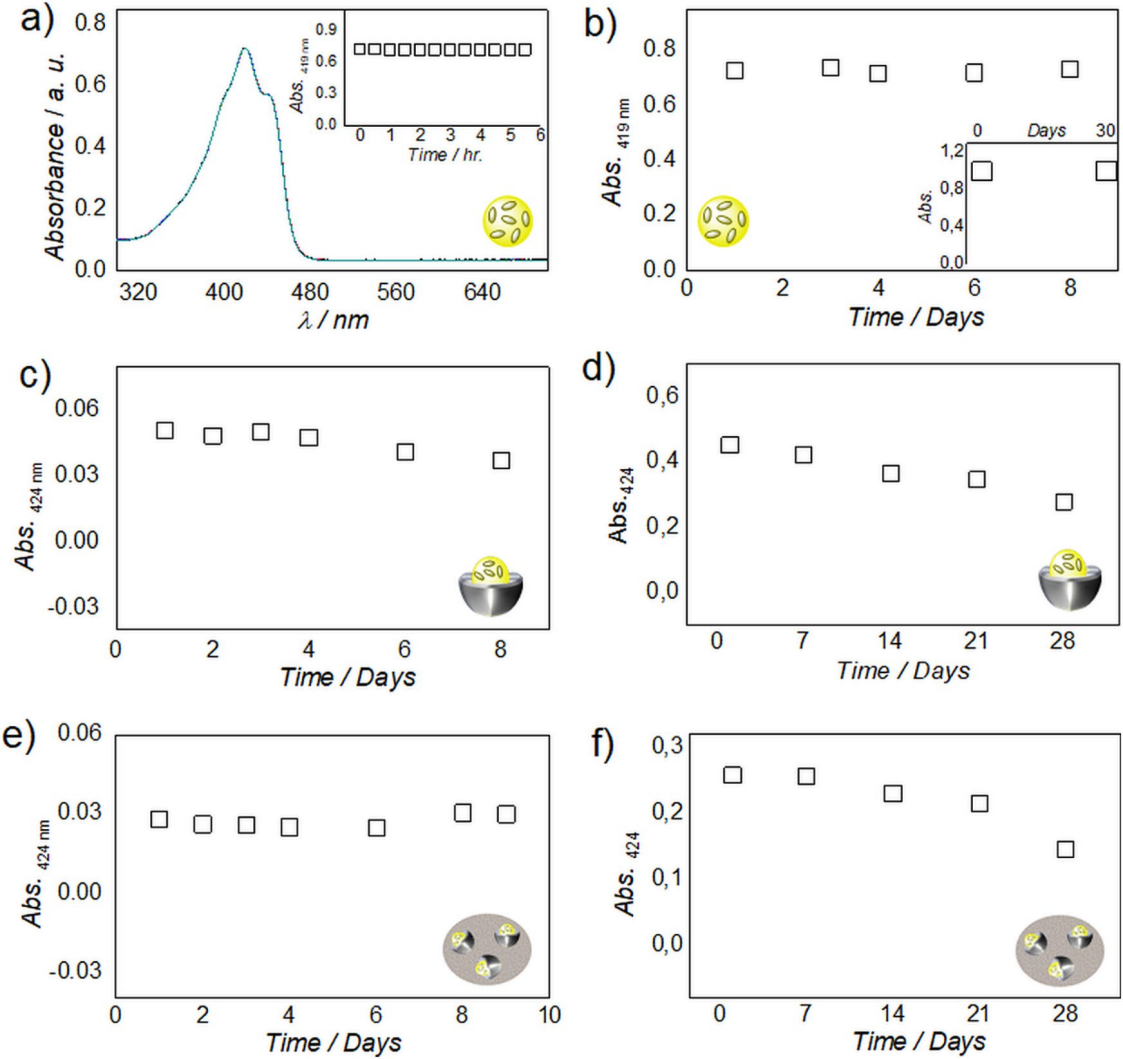
(**a**) UV–Vis spectra of curcumin in olive oil upon continuous irradiation for 5.5 h (inset showing no change in absorbance at 419 nm after 5.5 h). (**b**) Absorbance (419 nm) versus time (days) profile of curcumin in olive oil for 8 days and 30 days (inset) (**c**,**d**) Absorbance (at 424 nm) versus time (days) profile of curcumin in olive oil-PF127-water nanoemulsion (Cur-NE) for 8 (**c**) and 28 days (**d**). **e**–**f**, Absorbance (at 424 nm) versus time (days) profile of curcumin in gelatin encapsulated olive oil-PF127-water nanoemulsion (G-Cur-NE) for 9 (**e**) and 28 days (**f**).

The developed strategy was then studied for the stabilization of curcumin in antioxidant pools^31,32^ of olive oil in NEs encapsulated by gelatin for around a week and a month separately (Fig. 5). Firstly, curcumin was dissolved in olive oil (0.5 mg g^−1^) at 50 °C for 24 h. Then, its chemical stability was studied as a function of time by UV–Vis spectroscopy.

Curcumin remained perfectly stable against continuous UV–Vis irradiation for 5.5 h on day 1 (Fig. 5a) and did not show any decrease in peak at 419 nm (Figs. 5b and S12a from ESI) for a week and a month (inset Figs. 5b and S12b from ESI), thus indicating its stability in olive oil. Olive oil containing curcumin was used to prepare nanoemulsions (Cur-NE) and gelatin encapsulated nanoemulsions (G-Cur-NE), as shown in Fig. S1b from ESI. The chemical stability of Cur in Cur-NE and G-Cur-NE was studied also using UV–Vis spectroscopy (Figs. 5c–f & S12c–f from ESI). Curcumin showed just 26% degradation in Cur-NE after 8 days (Figs. 5c & S12c from ESI), whereas it remained perfectly stable in G-Cur-NE, hence indicating the protective shielding of gelatin against degradation for around a week (Figs. 5e & S12e from ESI). In a separate study performed during one month, a degradation rate of curcumin around 26% was observed in Cur-NE (Figs. 5d & S12d from ESI) after 3 weeks compared to just 16% in G-Cur-NE (Figs. 5f & S12f from ESI). This result was followed by 38% degradation of curcumin in Cur-NE compared to 44% in G-Cur-NE) in the fourth week (Fig. 5d,f). The higher degradation in gelatin in the fourth week is not a surprising result since proteins can retain stability in solution at 4–8 °C, for no longer than one or two weeks. However, this issue can be sorted by preparing a fresh solution of gelatin for encapsulation of Cur-NE which retained > 60% of the active form of curcumin after storage for 4 weeks.

### Cellular uptake, viability and anticancer activity of the Cur-NE and G-Cur-NE

**Figure 6:**
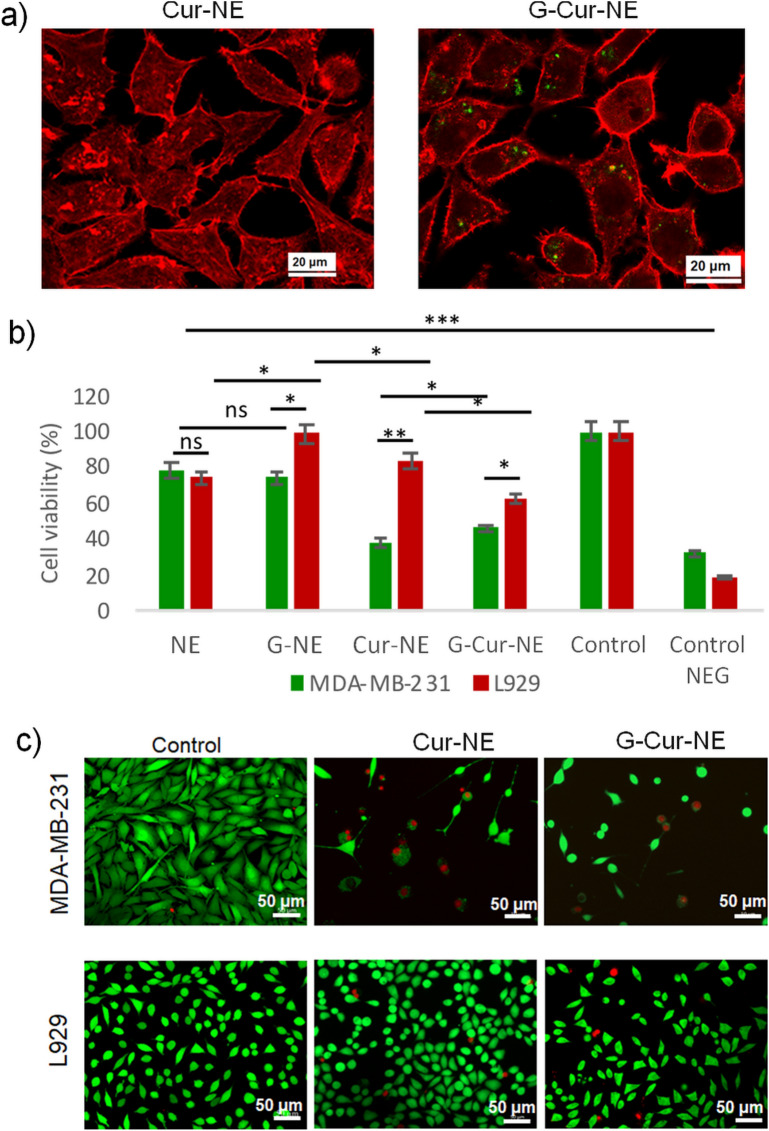
(**a**) Uptake of Cur-NE and G-Cur-NE on L929 cells. Red channel f-actin and Green channel emission of curcumin. Evaluation of the NE, G-NE, Cur-NE and G-Cur-NE effect on the metabolism and membrane integrity of MDA-MD-231 and L929 cells. (**b**) Cellular viability of both cell lines. Data represents three independent assays with six replicates each. *p-value < 0.05, **p-value < 0.01, ***p-value < 0.001 and ns = non-significant. (**c**) Membrane integrity of both cells after incubation with Cur-NE and G-Cur-NE Green channel: live cells (membrane stained with Calcein-AM), Red channel: dead cells (nucleus stained with PI).

Being widely used for biocompatibility screening assays, L929 fibroblasts were chosen as healthy cells having the effect of these NE compared with breast cancer fibroblasts (MDA-MB-231) as a proof of concept. The cellular uptake of curcumin was tested on L929 using Cur-NE and G-Cur-NE as delivery vehicles (Fig. 6a). Interestingly we observed fluorescence of curcumin only inside the L929 cells incubated with G-Cur-NE (Fig. 6a). It indicates that curcumin can be efficiently delivered into the cells when nanoemulsions were coated with the gelatin. The effectiveness of these vehicles (Cur-NE and G-Cur-NE) on the delivery and activity of curcumin was evaluated MDA-MB-231 and compared with the L929 as presented in Figs. 6, c and S13, S14 from ESI. The cell’s viability (Fig. 6b) was assessed by analyzing the proliferation of cells for 72 h. upon incubation with various delivery vehicles.

The metabolic activity of L929 cells in G-NE, Cu-NE and G-Cur-NE was found to be higher indicating their preferential inhibition effect on MDA-MB-231 cells. These results were complemented by the live/dead assay wherein both NE and G-NE (Fig. S13 from ESI) showed no impair on the membrane integrity of L929 cells (exhibited by the green staining) but some disruption on MDA-MB-231 cells (red staining). The introduction of curcumin (Fig. S14 from ESI) showed significant disruption of MDA-MB-231 cell membrane (dead cells stained in red) with the decrease in cell density. The round shape observed for most cells (compared to control) suggests detachment after being dead. L929 cells, on the other hand, showed signals of reduction on the cell density but with low cellular death and retained similar shape to those of control (Fig. S14 from ESI).

## Discussion

Although olive oil has been extensively used as water in oil microemulsion (W/O) for various applications, there are few reports of its utility as oil in water (O/W) micro/ nanoemulsion^50–54^. These microemulsion were prepared using either co-surfactants or a number of chemical stabilizers using highly energy intensive procedures like ultrasonication and temperature induced phase inversion etc. However, our procedure of olive oil in water nanoemulsion preparation is highly energy efficient wherein nanoemulsion is prepared just by 15 min. of rotation without any cosurfactant. Moreover, unlike the previously published reports^50–54^. we have also presented first visual evidence (TEM image) of olive oil sitting in the micellar cavity of the surfactant, PF 127 (Fig. 3c–e). This is also the first report on olive oil in water nanoemulsion using PF 127 as surfactant. The time dependent DLS analysis of NE showed no ripening of micelles in NE solution stored at 4–8 °C for a week and a slight ripening effect when stored for a month (Fig. 4a,b), which indicates the preparation of stable dispersions for NE. The curcumin was previously studied for its stability in oils including extra virgin olive oil in the form of microemulsion. However, the microemulsion was prepared at toxic concertation of tween 80 (30%) as surfactant and 800 mM of NaCl as electrolyte using complex temperature phase inversion temperature method. This is because, it was impossible to prepare transparent O/W microemulsions at lower concentration of Tween 80 due to high hydrophobicity of oils. Moreover, curcumin also showed poor chemical stability in the reported microemulsion with time^54^. Contrary to this^54^ we have used a cytocompatible surfactant PF127 at a very low concentration of 2% so that the develop vehicles can be demonstrated for in vivo delivery of curcumin. The native olive oil showed perfect antioxidation behaviour against the chemical degradation of curcumin for a month (Fig. 5a,b). But, like tween 80 based ME^54^ curcumin showed slight degradation in our olive oil-PF127-water NE after 4 days (Fig. 5c). However, the degradation is just 24% when stored for 3 weeks (Fig. 5d), therefore justifying the longevity of the antioxidation action of olive oil in NE. The curcumin degradation could be improved upon coating the Cur-NE with gelatin when studied for 9 days (Fig. 5e). The stability of curcumin in G-Cur-NE is better than in water (t_1/2_ ~ 20 min.) and recently reported aqueous IL complex (t_1/2_ ~ 260 min.)^11^, therefore justifying the protective action of olive oil and gelatin against the drug autoxidation. Unlike the Cur-NE the gelatin coated Cur-NE showed good cellular uptake as observed from the fluorescence of curcumin (Fig. 6a). It is because, unlike Cur-NE the gelatin present at the surface of G-Cur-NE might have acted like a carrier protein to diffuse curcumin into the cell cytoplasm^32,33^. On the other hand, in Cur-NE the NE is composed of surfactant and olive oil containing triglycerides, which can assimilate into the cell membrane along with curcumin^2,12^. Hence showing poor diffusion of curcumin into the cytoplasm. Therefore, our conceptualization of using protein as surface coat for efficient transit of colloidal delivery vehicles through the cell membrane is vindicated. As a proof of concept, anticancer testing on MDA-MD-231 cells both Cur-NE and G-Cur-NE mitigated the cancers cells as seen from Fig. 6c. Moreover, even without curcumin the NE showed mitigation of cancer cells (Fig. S13 from ESI). This behavior indicates the anticancer behaviour of olive oil against MDA-MD-231 cells^37^. Poor cell viability of L929 cells in the presence of Tween 80 micelles (Control negative) which generally used in NE preparation for curcumin delivery also justifies the use of PF 127 as cyto-compatible surfactant for NE preparation herein. The mechanism for anticancer effect of curcumin is still disputed and many reports have hypothesized the better uptake of curcumin by cancer cells as compared to healthy cells on grounds of membrane constitution as the reason^55–60^. Herein, opposite to MDA-MB-231 cells, the L929 cells did not show any change in cell morphology even after curcumin internalization (Fig. 6c). Therefore, the 20% reduction in the metabolic activity of L929 cells can be due to growth inhibition (cell cycle arrest)^52^ in opposition to the apoptotic effect of curcumin on MDA-MB-231 cells by a sequence of events reported elsewhere^61–63^. Unravelling the mechanism of action of G-Cur-NE against healthy versus cancer cells will be objective of future work.

### Summary and conclusions

In this work, a simple and robust strategy of protein encapsulation of olive oil-in-water nanoemulsions was developed; 1) to avoid the autoxidation of curcumin and 2) to increase its cellular uptake. Olive oil perfectly retained the structural integrity of curcumin against continuous UV–Vis irradiation through time. Its aqueous formulation with PF 127 as nanoemulsion also protected curcumin against autoxidation possibly by scavenging of O_2_˙ˉ, RO˙ and OH˙ by the antioxidants present in olive oil. Encapsulation of Cur-NE formulation with protein gelatin further enhanced its resistance against auto-oxidation along with an increased cellular uptake. A clear structural evidence of gelatin encapsulated NE is obtained from TEM wherein oil droplet could be seen sitting in the micellar pocket of PF 127. The G-Cur-NE showed a preferential inhibition effect on MDA-MB-231 breast cancer cells tested as proof of concept, thus giving insights of its anticancer activity. The same system will be tested on skin cancer cells and others in our futurely work to enlarge the database associated and to futurely serve as basis for the production of new biomaterials. The design principles developed in this work provide a new alternative route to deliver oxidation prone drugs for selective action against infected cells.

## Supplementary information

Supplementary Figures.

## Data Availability

All the data of this manuscript is available in the main text and supplementary information. Additional data of original data files can be accessed on a reasonable request to the corresponding author by mail.

## References

[1] Zheng B, Zhang X, Peng S, McClements DJ (2019). Impact of curcumin delivery system format on bioaccessibility: nanocrystals, nanoemulsion droplets, and natural oil bodies. Food Funct..

[2] Barry J (2009). Determining the effects of lipophillic drugs on membrane structure by solid-state NMR spectroscopy-the case of the antioxidant curcumin. J. Am. Chem. Soc..

[3] Ammon HP, Wahl MA (1991). Pharmacology of curcuma longa. Planta Med..

[4] Gupta SC (2011). Multitargeting by curcumin as revealed by molecular interaction studies. Nat. Prod. Rep..

[5] Salem M, Rohani S, Gillies ER (2014). Curcumin, a promising anti-cancer therapeutic: a review of its chemical properties, bioactivity and approaches to cancer cell delivery. RSC Adv..

[6] Kocaadam B, Sanlier N (2017). Curcumin, an active component of turmeric (curcuma longa), and its effects on health. Crit. Rev. Food Sci. Nutr..

[7] Kharat M, McClements DJ (2019). Recent advances in colloidal delivery systems for nutraceuticals: a case study—delivery by design of curcumin. J. Colloid Interface Sci..

[8] Vecchione R, Quagliariello V, Calabria D, Calcagno V, Luca ED, Laffaioli R, Netti PA (2016). Curcumin bioavailability from oil in water nano-emulsions: in vitro and in vivo study on the dimensional, compositional and interactional dependence. J. Control. Release.

[9] Anand P, Kunnumakkara AB, Newman RA, Aggarwal BB (2007). Bioavailability of curcumin: problems and promises. Mol. Pharm..

[10] Nagahama K (2016). Discovery of a new function of curcumin which enhances its anticancer therapeutic potency. Sci. Rep..

[11] Chowdhury MR (2019). Development of a novel ionic liquid-curcumin complex to enhance its solubility, stability, and activity. Chem. Commun..

[12] Wang D (2008). Liposome-encapsulated curcumin suppresses growth of head and neck squamous cell carcinoma in vitro and in xenografts through the inhibition of nuclear factor kappaB by an AKT-independent pathway. Clin. Cancer Res..

[13] Barick KC (2016). Pluronic stabilized Fe_3_O_4_ magnetic nanoparticles for intracellular delivery of curcumin. RSC Adv..

[14] Gou M (2011). Curcumin-loaded biodegradable polymeric micelles for colon cancer therapy in vitro and in vivo. Nanoscale.

[15] Gao X (2013). Improving the anti-colon cancer activity of curcumin with biodegradable nano-micelles. J. Mater. Chem. B.

[16] Hu LD (2012). Preparation and enhancement of oral bioavailability of curcumin using microemulsions vehicle. J. Agric. Food Chem..

[17] Niu YM (2012). Effects of curcumin concentration and temperature on the spectroscopic properties of liposomal curcumin. J. Agric. Food Chem..

[18] Huang M (2019). Liposome co-encapsulation as a strategy for the delivery of curcumin and resveratrol. Food Funct..

[19] Huang Q (2014). Coating of carboxymethyl dextran on liposomal curcumin to improve the anticancer activity. RSC Adv..

[20] Araiza-Calahorra A, Akhtar M, Sarkar A (2018). Recent advances in emulsion-based delivery approaches for curcumin: from encapsulation to bioaccessability. Trends Food Sci. Technol..

[21] Xu GR, Wang CN, Yao P (2017). Stable emulsion produced from casein and soy polysaccharide compacted complex for protection and oral delivery of curcumin. Food Hydrocolloids.

[22] Zheng BJ, Peng SF, Zhang XY, McClements DJ (2018). Impact of delivery system type on curcumin bioaccessibility: comparison of curcumin-loaded nanoemulsions with commercial curcumin supplements. J. Agric. Food Chem..

[23] Nikolic I (2018). Curcumin-loaded low-energy nanoemulsions as a prototype ofmultifunctional vehicles for different administration routes: physicochemical andin vitropeculiarities important for dermal application. Int. J. Pharm..

[24] Nikolic I (2020). Microstructure and biopharmaceutical performances of curcumin-loaded low-energy nanoemulsions containing eucalyptol and pinene: terpenes’ role overcome penetration enhancement effect ?. Eur. J Pharm. Sci..

[25] Kumar R, Uppal S, Kaur K, Mehta SK (2020). Curcumin nanoemulsion as a biocompatible medium to study the metal ion imbalance in a biological system. J. Mol. Liq..

[26] Weiss J (2008). Solid lipid nanoparticles as delivery systems for bioactive food components. Food Biophys..

[27] Wang TR, Ma XY, Lei Y, Luo YC (2016). Solid lipid nanoparticles coated with cross-linked polymeric double layer for oral delivery of curcumin. Colloids Surf. B-Biointerfaces.

[28] Ramalingam P, Yoo SW, Ko YT (2016). Nanodelivery systems based on mucoadhesive polymer coated solid lipid nanoparticles to improve the oral intake of food curcumin. Food Res. Int..

[29] Kakkar V, Singh S, Singla D, Kaur IP (2011). Exploring solid lipid nanoparticles to enhance the oral bioavailability of curcumin. Mol. Nutr. Food Res..

[30] Ubeyitogullari A, Ciftci ON (2019). A novel and green nanoparticle formation approach to forming low-crystallinity curcumin nanoparticles to improve curcumin’s bioaccessibility. Sci. Rep..

[31] Jovanovic SV, Boone CW, Steenken S, Trinoga M, Kaskey RB (2001). How curcumin works preferentially with water soluble antioxidants. J. Am. Chem. Soc..

[32] Alberts, B. *et al*. Molecular biology of the cell. 4th edition, New York: *Garland Science*. ISBN-10: 0-8153-3218-1 (2002).

[33] Crosbya J, Crump MP (2012). The structural role of the carrier protein-active controller or passive carrier. Nat. Prod. Rep..

[34] Gordon ON, Luis PB, Sintim HO, Schneider C (2015). Unraveling curcumin degradation: autoxidation proceeds through spiroepoxide and vinylether intermediates en route to the main bicyclopentadione. J. Biol. Chem..

[35] Covas MI, Konstantinidou V, Fitó M (2009). Olive oil and cardiovascular health. J. Cardiovasc. Pharmacol..

[36] Serrano EMY, Moreno JL, Delgado FG, Miranda JL (2018). Extra virgin olive oil: more than a healthy fat. Eur. J. Clin. Nutr..

[37] Owen RW (2004). Olives and olive oil in cancer prevention. Eur. J. Cancer Prev..

[38] Khattak SF, Bhatia SR, Roberts SC (2005). Pluronic F127 as a cell encapsulation material: utilization of membrane-stabilizing agents. Tissue Eng..

[39] Sasaki Y, Oshikawa M, Bharmoria P, Kouno H, Hayashi-Takagi A, Sato M, Ajioka I, Yanai N, Kimizuka N (2019). Near-infrared optogenetic genome engineering based on photon-upconversion hydrogels. Angew. Chem. Int. Ed..

[40] Kozlov MY, Melik-Nubarov NS, Batrakova EV, Kabanov AV (2000). Relationship between pluronic block copolymer structure, critical micellization concentration and partitioning coefficients of low molecular mass solutes. Macromolecules.

[41] Bharmoria P, Hisamitsu S, Nagatomi H, Ogawa T, Morikawa M-A, Yanai N, Kimizuka N (2018). Simple and versatile platform for air-tolerant photon upconverting hydrogels by biopolymer-surfactant-chromophore co-assembly. J. Am. Chem. Soc..

[42] Gouvinhas I, Machado J, Gomes S, Lopes J, Martins-Lopes P, Barros AIRNA (2014). Phenolic composition and antioxidant activity of monovarietal and commercial portuguese olive oils. J. Am. Oil Chem. Soc..

[43] Wu H-L, Wang G-H, Xiang W-Z, Li T, He H (2016). Stability and antioxidant activity of food-grade Phycocyanin isolated from *Spirulina Platensis*. Int. J. Food Prop..

[44] Cai Y, Luo Q, Sun M, Corke H (2004). Antioxidant activity and phenolic compounds of 112 traditional Chinese medicinal plants associated with anticancer. Life Sci..

[45] Re R, Pellegrini N, Proteggente A, Pannala A, Yang M, Rice-Evans C (1999). Antioxidant activity applying an improved ABTS radical cation decolorization assay. Free Rad. Biol. Med..

[46] Papadimitriou V, Sotiroudis TG, Xenakis A (2007). Olive oil microemulsions: enzymatic activities and structural characteristics. Langmuir.

[47] Papadimitriou V, Sotiroudis TG, Xenakis A (2005). Olive oil microemulsions as a biomimetic medium for enzymatic studies: oxidation of oleuropein. J. Am. Oil Chem. Soc..

[48] McClements DJ (2012). Nanoemulsions versus microemulsions: terminology, differences, and similarities. Soft Matter.

[49] Bastos H, Bento R, Schaeffer N, Coutinho JAP, Pérez-Sánchez G (2020). Using coarse-grained molecular dynamics to rationalize biomolecule solubilization mechanisms in ionic liquid-based colloidal systems. Phys. Chem. Chem. Phys..

[50] Kuraya E (2016). Direct analysis of lipophilic antioxidants of olive oils using bicontinuous microemulsions. Anal. Chem..

[51] Mehmood T, Ahmad A, Ahmed A, Ahmed Z (2017). Optimization of olive oil-based O/W nanoemulsions prepared through ultrasonic homogenization: a response surface methodology approach. Food Chem..

[52] Salam JA, Das N (2013). Enhanced biodegradation of lindane using oil-in-water bio-microemulsion stabilized by biosurfactant produced by a new yeast strain. J. Microbiol. Biotechnol..

[53] Chaiyana W, Leelapornpisid P, Phongpradist R, Kiattisin K (2016). Enhancement of antioxidant and skin moisturizing effects of olive oil by incorporation into microemulsions. Nanomater. Nanotechnol..

[54] Calligaris S (2017). Development of transparent curcumin loaded microemulsions by phase inversion temperature (PIT) method: effect of lipid type and physical state on curcumin stability. Food Biophys..

[55] Liu HT, Ho YS (2018). Anticancer effect of curcumin on breast cancer and stem cells. Food Sci. Hum. Wellness.

[56] Sordillo PP, Helson L (2015). Curcumin and cancer stem cells: curcumin has asymmetrical effects on cancer and normal stem cells. Anticancer Res..

[57] Dai X (2017). Nano-formulated curcumin accelerates acute wound healing through Dkk-1-mediated fibroblast mobilization and MCP-1-mediated anti-inflammation. NPG Asia Mater..

[58] Sharma M, Sahu K, Singh SP, Jain B (2018). Wound healing activity of curcumin conjugated to hyaluronic acid: in vitro and in vivo evaluation. Artif. Cells Nanomed. Biotechnol..

[59] Sarkar N, Bose S (2019). Liposome-encapsulated curcumin-loaded 3-D printed scaffold for bone tissue engineering. ACS Appl. Mater Interfaces.

[60] Kudina O (2015). Invertible micellar polymer nanoassemblies target bone tumor cells but not normal osteoblast cells. Future Sci. OA.

[61] Giordano A, Tommonaro G (2019). Curcumin and cancer. Nutrients.

[62] Song X, Zhang M, Dai E, Luo Y (2019). Molecular targets of curcumin in breast cancer. Mol. Med. Rep..

[63] Vallianou NG, Evangelopoulos A, Schizas N, Kazazis C (2015). Potential anticancer properties and mechanisms of action of curcumin. Anticancer Res..

