# Supplementary material for: Protein-olive oil-in-water nanoemulsions as encapsulation materials for curcumin acting as anticancer agent towards MDA-MB-231 cells
Source: Sci Rep. 2021 Apr 27;11:9099. doi: 10.1038/s41598-021-88482-3 (PMC8079396; doi:10.1038/s41598-021-88482-3)
Supplement: Supplementary file 1 — Supplementary Figures. [file 41598_2021_88482_MOESM1_ESM.docx]

**Protein-Olive Oil-in-Water Nanoemulsions as Encapsulation Materials for Curcumin Acting as Anticancer Agent towards MDA-MB-231 Cells**

Pankaj Bharmoria^‡^*^a^* Meena Bisht^‡^*^a^* Maria C. Gomes^‡^*^a^* Margarida Martins,*^a^* Márcia C. Neves, *^a^* João F. Mano, *^a^* Igor Bdikin *^b^*, João A. P. Coutinho*^a^* and Sónia P. M. Ventura**^a^*

*^a^CICECO-Aveiro Institute of Materials, Department of Chemistry, University of Aveiro, 3810-193, Portugal. Email:* [*spventura@ua.pt*](mailto:spventura@ua.pt)

*^b^*TEMA, Department of Mechanical Engineering, University of Aveiro, 3810-193 Aveiro, Portugal.

*^c^*Department of Applied Chemistry, Chalmers University of Technology, Kemivägen 4,412 96 Gothenburg, Sweden.

^‡^PB, MB and MCG have contributed equally towards this work.

*Electronic Supplementary Information (ESI)*


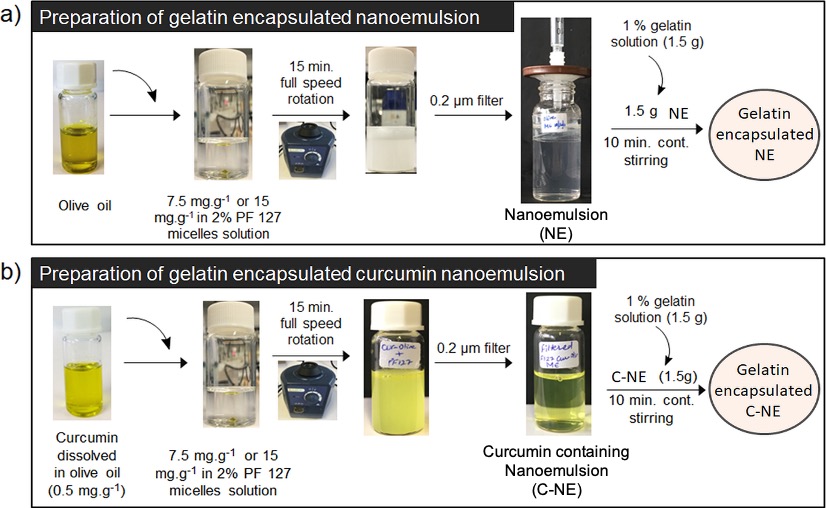


**Figure S1.** **a**, Preparation of gelatin encapsulated nanoemulsion (G-NE). **b,** Preparation of gelatin encapsulated curcumin containing nanoemulsion (G-Cur-NE).


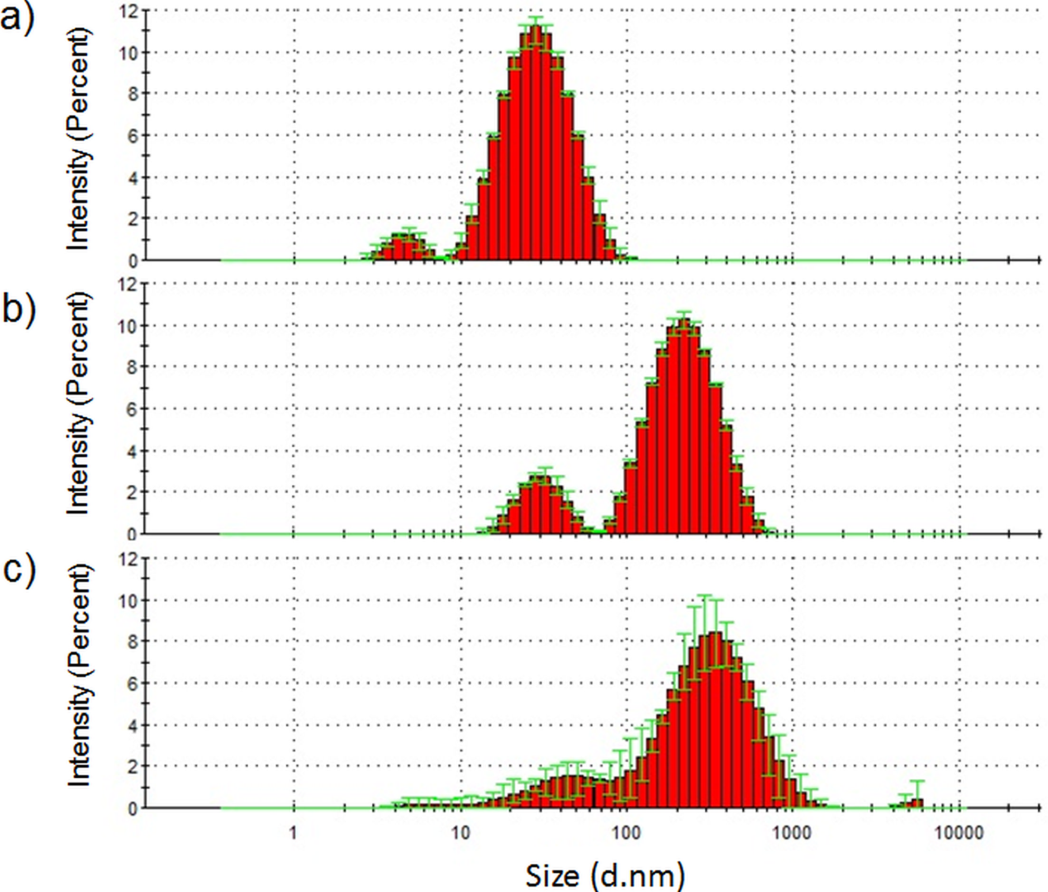


**Figure S2.** Hydrodynamic diameter profiles of **a**, PF 127 micelles; **b**, olive oil-PF 127-water nanoemulsion (NE) and **c**, gelatin encapsulated olive oil-PF 127-water nanoemulsion (G-NE).


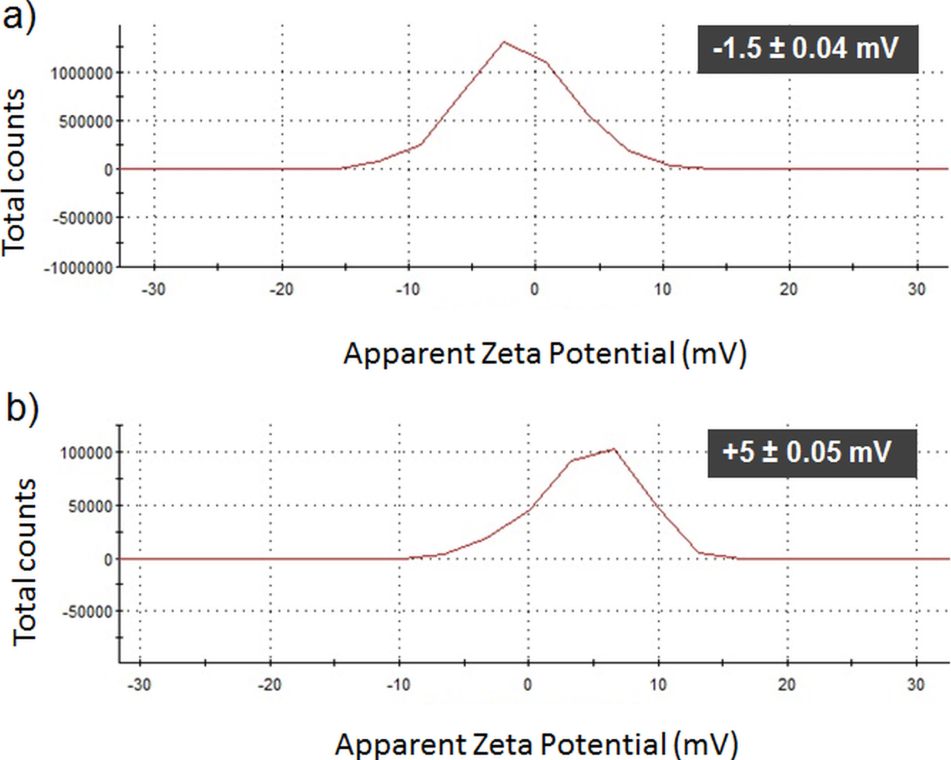


**Figure S3.** Apparent zeta potential profiles of **a**, olive oil-PF 127-water nanoemulsion (NE) and **b**, gelatin encapsulated olive oil-PF 127-water nanoemulsion (G-NE).


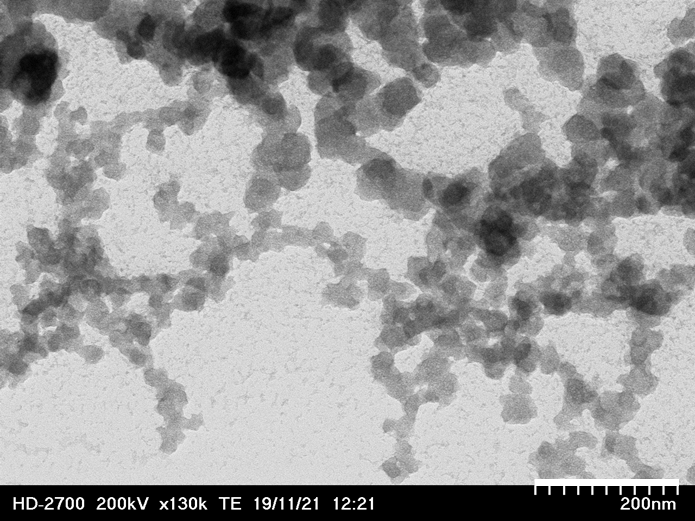


**Fig. S4**. TEM image of PF 127 micelles.


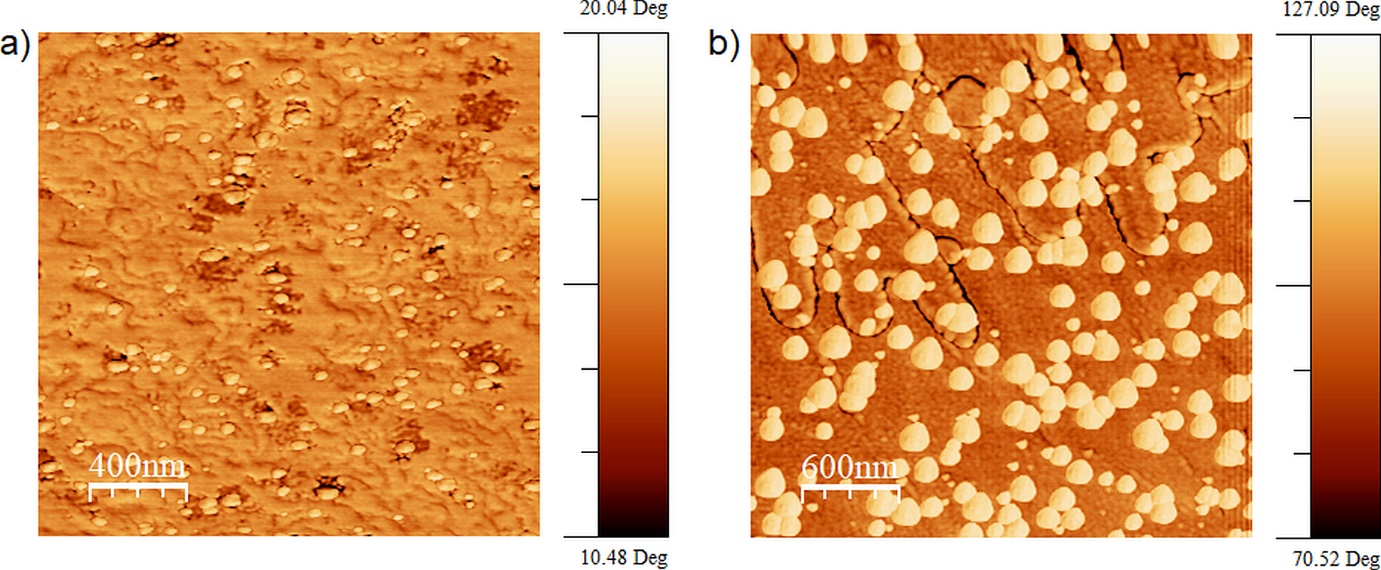


**Fig. S5**. Phase mode AFM images of a) PF127 micelles and b) Olive oil-PF 127-water NE


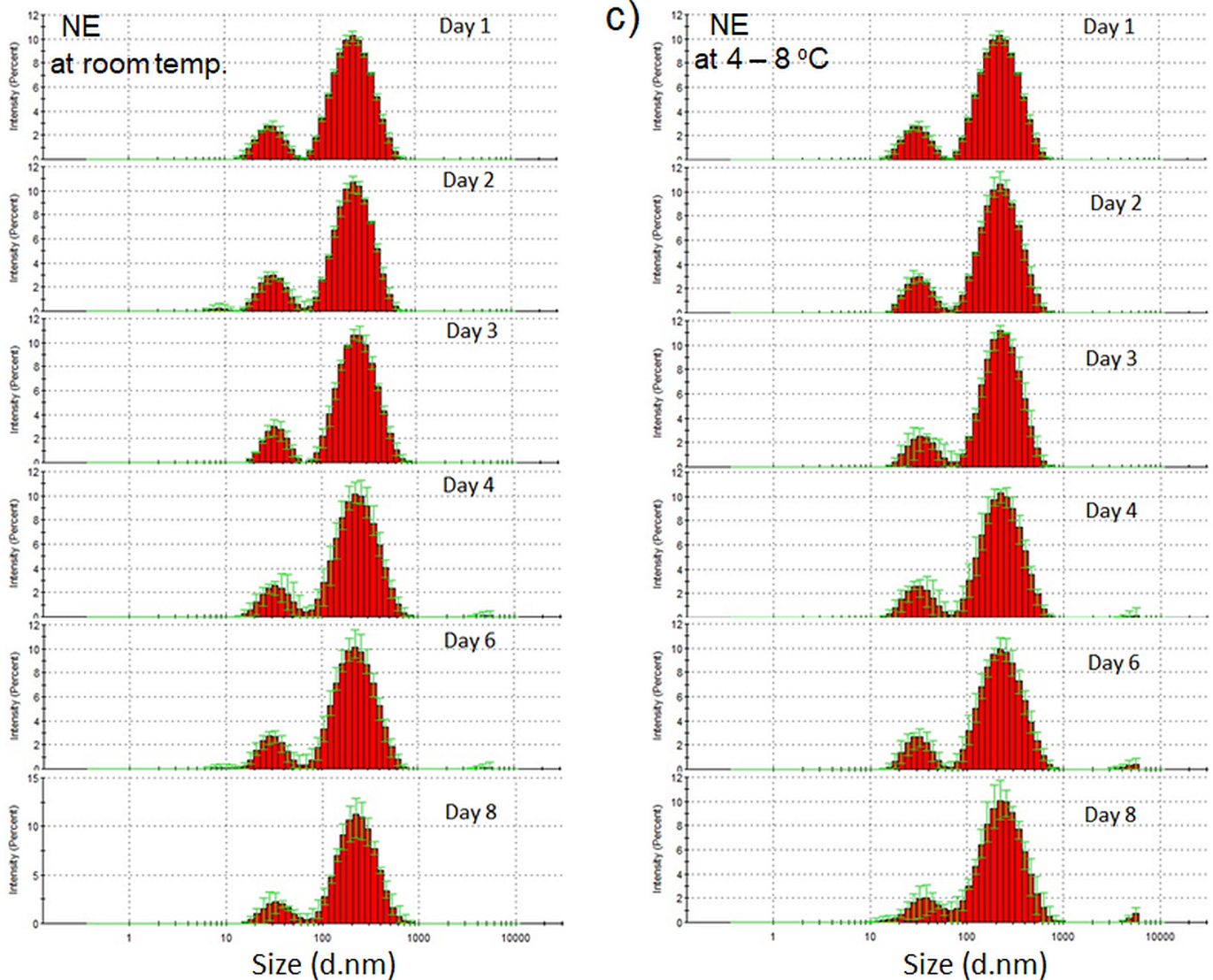


**Figure S6.** Comparative hydrodynamic diameter profiles of olive oil-PF 127-water nanoemulsions with time (days) for 8 days, **a)** stored at room temperature and **b)** stored at 4-8 ^o^C in a refrigerator.


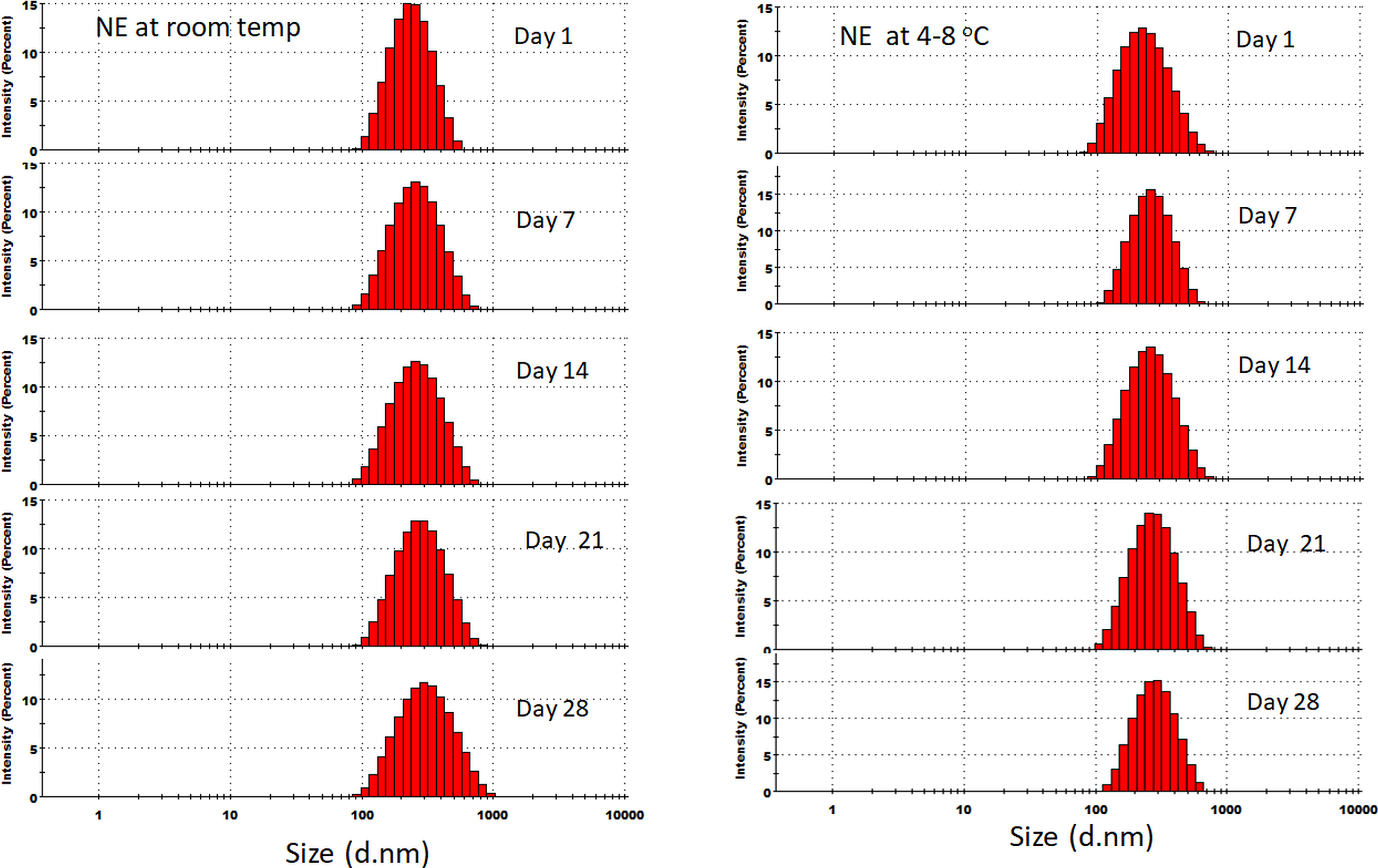


**Figure S7.** Comparative hydrodynamic diameter profiles of olive oil-PF 127-water nanoemulsions with time (days) for 28 days, **a)** stored at room temperature and **b)** stored at 4-8 ^o^C in a refrigerator.


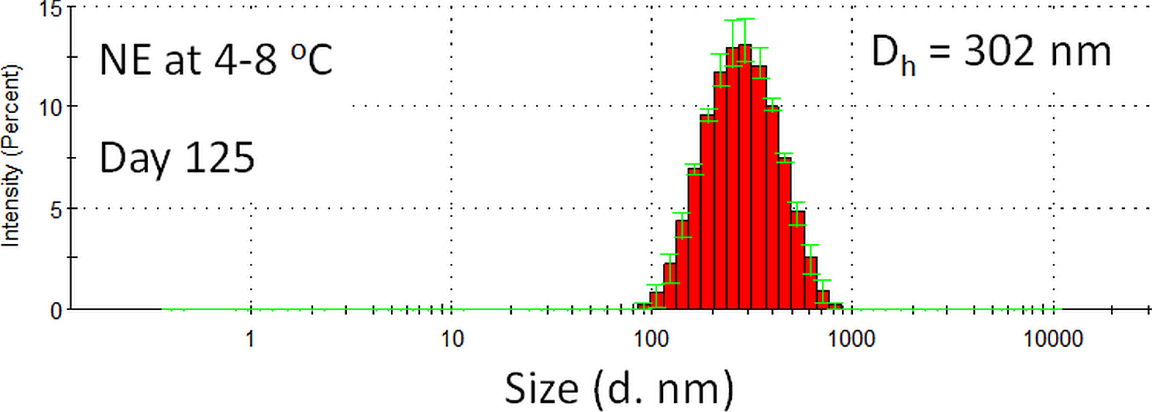


**Figure S8.** Hydrodynamic diameter profiles of olive oil-PF 127-water nanoemulsions with time (days) for 125 days, stored at 4-8 ^o^C in a refrigerator.


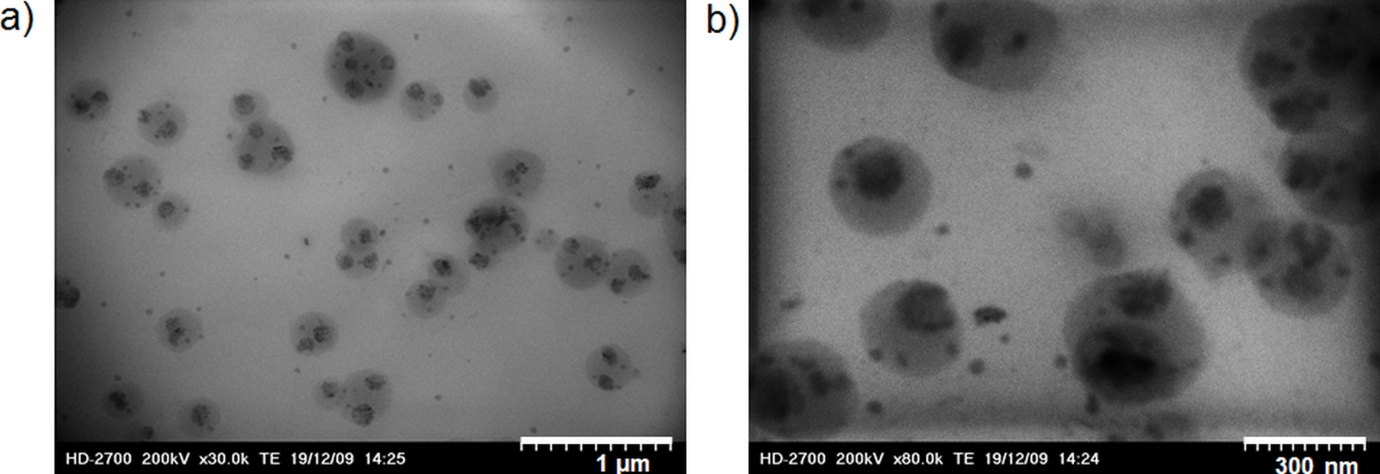


**Figure S9.** Transmission electron microscopy images of gelatin encapsulated olive oil-PF 127-water nanoemulsions (G-NE) **a**, scale bar 1 µm and **b**, scale bar 300 nm.


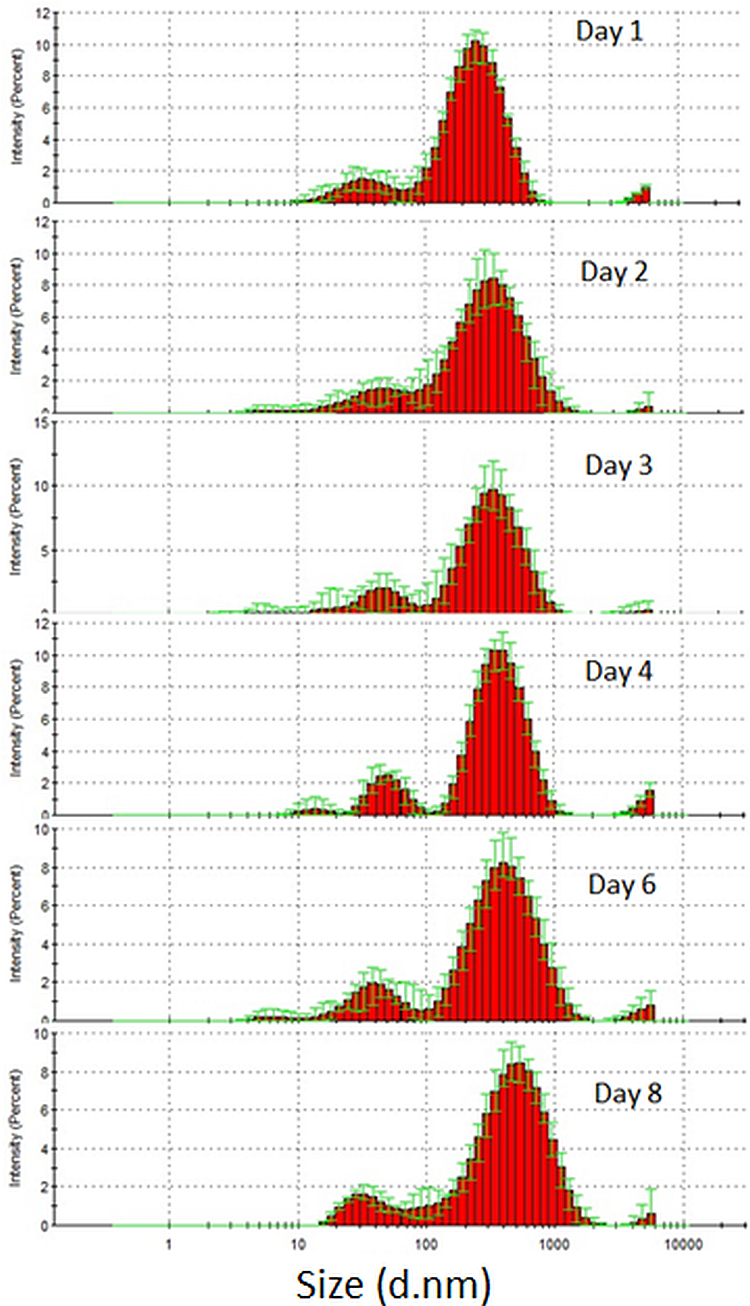


**Figure S10.** Comparative hydrodynamic diameter profiles of gelatin encapsulated olive oil-PF 127-water nanoemulsions (G-NE) with time (days) stored at 4-8 ^o^C in a refrigerator for 8 days.


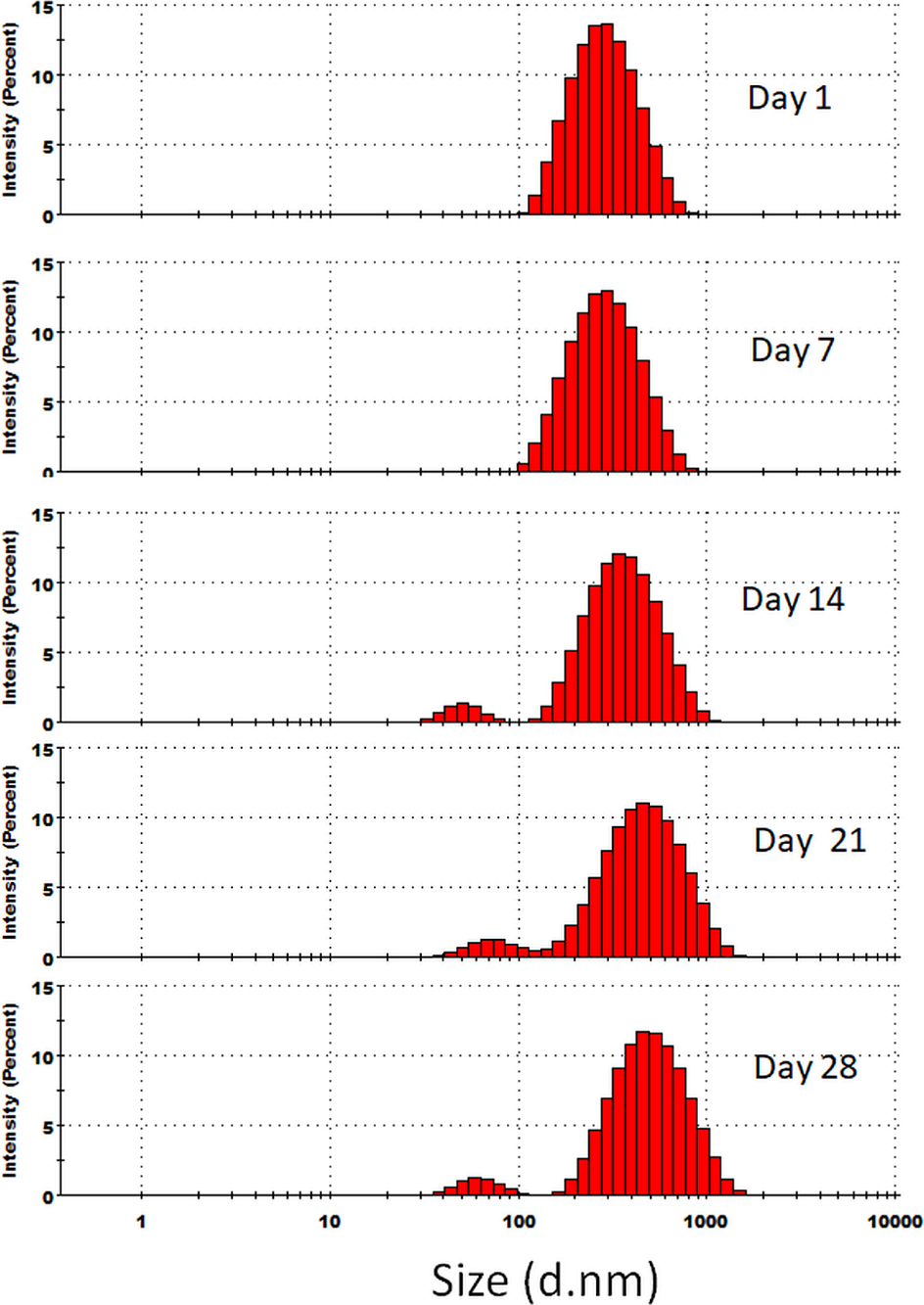


**Figure S11.** Comparative hydrodynamic diameter profiles of gelatin encapsulated olive oil-PF 127-water nanoemulsions (G-NE) with time (days) stored at 4-8 ^o^C in refrigerator for 28 days.


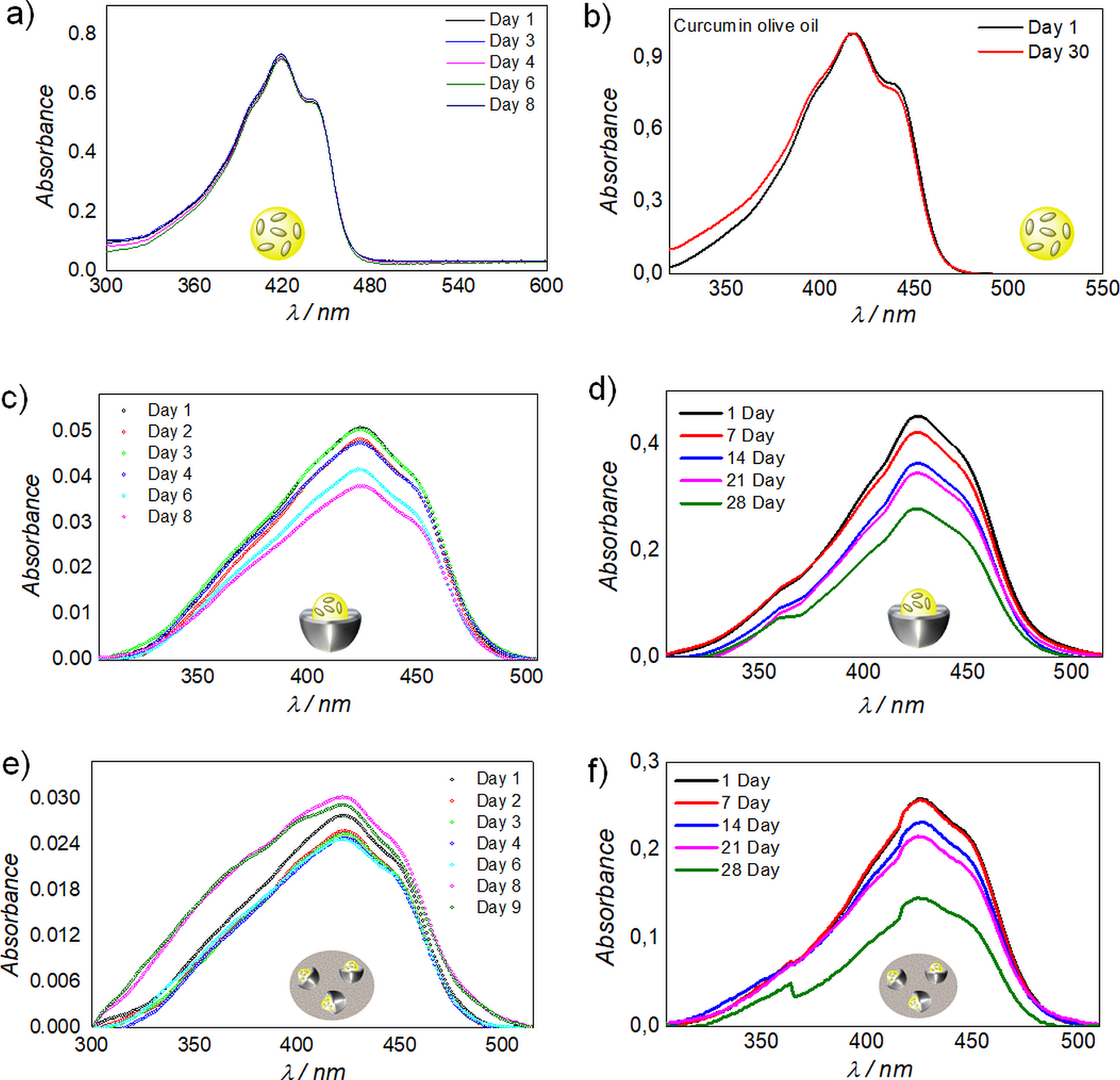


**Figure S12.** Time (days) dependent UV-Vis absorption spectra of curcumin dissolved in, **a-b**) in olive oil for **a)** 8 days and **b)** 30 days. **c-d)** in olive oil-PF 127-water nanoemulsion (Cur-NE) for **c**) 8 days and **d**) 28 days. **e-f)** in gelatin encapsulated olive oil-PF 127-water nanoemulsion (G-Cur-NE) for **e**, 9 days and **f**) 28 days.


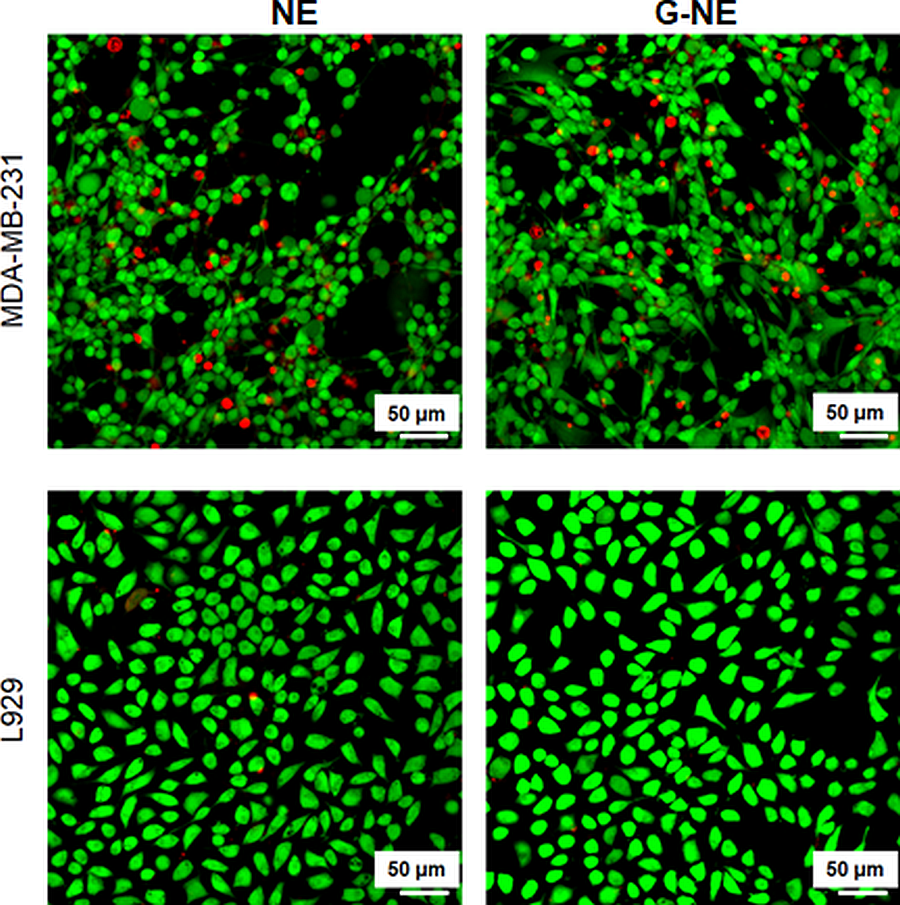


**Figure S13.** Membrane integrity of both cells after incubation with NE and G-NE Green channel: live cells (membrane stained with Calcein-AM), Red channel: dead cells (nucleus stained with PI).


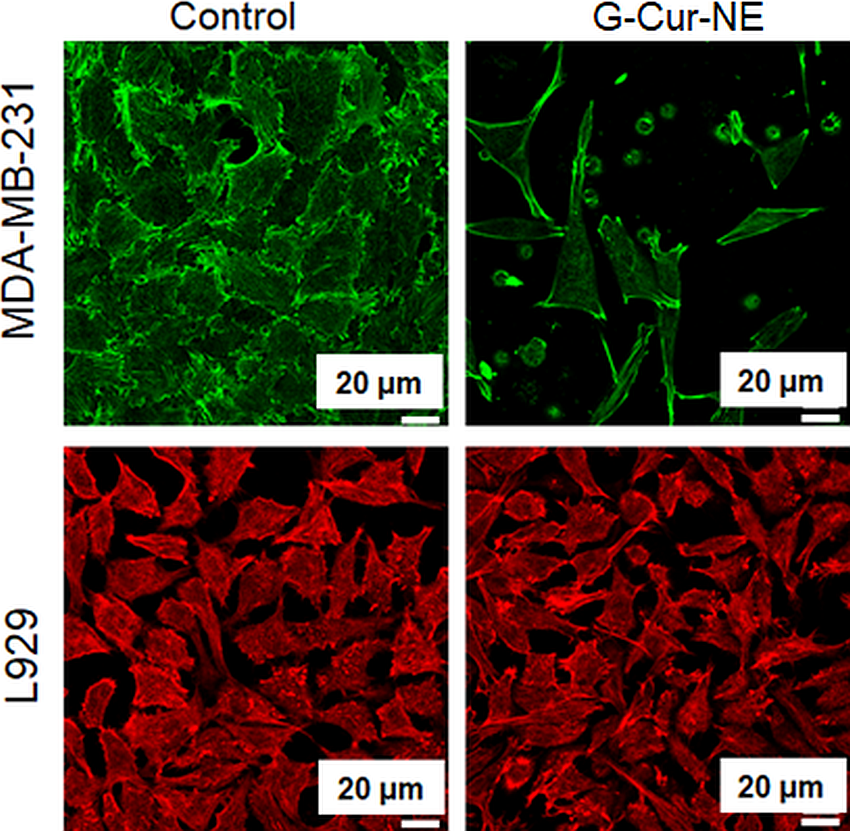


**Figure S14.** Morphology of both cells after incubation with G-Cur-NE. Both green and red channel are f-actin.
